# Comparative genetics of scyphozoan species reveals the geological history and contemporary processes of the Mediterranean Sea

**DOI:** 10.1002/ece3.7834

**Published:** 2021-07-08

**Authors:** Gur A. Mizrahi, Eli Shemesh, Avia Mizrachi, Dan Tchernov

**Affiliations:** ^1^ Department of Marine Biology The Leon H. Charney School of Marine Sciences University of Haifa Haifa Israel; ^2^ Morris Kahn Marine Research Station Environmental Geochemistry Lab. Leon H. Charney School of Marine Sciences Haifa University Haifa Israel; ^3^ Plant and Environmental Sciences Department Weizmann Institute of Science Rehovot Israel

**Keywords:** clock tree, invasive species, jellyfish, Lessepsian migration, Mediterranean Sea, Red Sea

## Abstract

Jellyfish are useful genetic indicators for aquatic ecosystems as they have limited mobility and are highly exposed to the water column. By using comparative genomics and the molecular clock (timetree) of *Rhizostoma pulmo*, we revealed a divergence point between the East and West Mediterranean Sea (MS) populations that occurred 4.59 million years ago (mya). It is suggested that the two distinct ecological environments we know today were formed at this time. We propose that before this divergence, the highly mixed Atlantic and Mediterranean waters led to the wide dispersal of different species including *R*. *pulmo*. At 4.59 mya, the Western and Eastern MS were formed, indicating the possibility of a dramatic environmental event. For the first time, we find that for the jellyfish we examined, the division of the MS in east and west is not at the Straits of Sicily as generally thought, but significantly to the east. Using genomics of the *Aurelia* species, we examined contemporary anthropogenic impacts with a focus on migration of scyphozoa across the Suez Canal (Lessepsian migration). *Aurelia* sp. is among the few scyphozoa we find in both the MS and the Red Sea, but our DNA analysis revealed that the Red Sea *Aurelia* sp. did not migrate or mix with MS species. *Phyllorhiza punctata* results showed that this species was only recently introduced to the MS as a result of anthropogenic transportation activity, such as ballast water discharge, and revealed a migration vector from Australia to the MS. Our findings demonstrate that jellyfish genomes can be used as a phylogeographic molecular tool to trace past events across large temporal scales and reveal invasive species introduction due to human activity.

## Graphical abstract

With the use of genetic tools, we revealed a divergence point between the East and West Mediterranean Sea populations that occurred 4.59 million years ago, and we propose that the western and eastern Mediterranean Sea were formed at that time. For the first time, we find that the division of the Mediterranean Sea in east and west is not at the Strait of Sicily, but significantly to the east. Aurelia sp. DNA analysis revealed that the Red Sea Aurelia sp. did not migrate or mix with Mediterranean Sea species. Phyllorhiza punctata results showed that this species was only recently introduced to the Mediterranean Sea from Australia due to human activity.

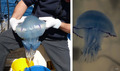

## 1 INTRODUCTION

Jellyfish are promising genetic indicators of the marine environment due to their unique body structure, limited mobility, and their prevalence in aquatic ecosystems. Jellyfish lack exoskeletons or hardened skin, their body consists of >95% water, and their wet weight contains <1% carbon, distinguishing them from other pelagic metazoans (Pitt et al., 2013). Due to this structure, jellyfish are highly exposed to the physio‐chemical conditions in the marine environment. In addition, their restricted movement and lack of fast swimming or active dispersal abilities force them to adapt to environmental fluctuations. Jellyfish have an ancient evolutionary lineage, with the oldest scyphozoans dated between 635 and 577 million years ago (mya) in the Neoproterozoic, the last era of the Precambrian (Van Iten et al., 2014). They successfully inhabit all oceans at all depths and in different niches. In this work, we aim to shed light on the puzzle of the Mediterranean Sea (MS) history. By examining the DNA of three jellyfish representative of Mediterranean macro‐jellyfish, some of the contemporary processes that are occurring in the MS may be better understood.

### 1.1 Oceanography

The Mediterranean Sea (MS) is a unique, semi‐enclosed marine system, which is naturally connected to the Atlantic Ocean via the Strait of Gibraltar. This waterway is 12.9 km wide and 286 m deep. A shallow sill at a depth of 350 m in the Strait of Sicily divides the MS into two basins, eastern and western. The Balearic, Ligurian, and Tyrrhenian subbasins constitute the western side of the Mediterranean, and the Ionian, Adriatic, Aegean, and Levantine the eastern. Seawater circulation in the MS is complex and divided into three major meridional and zonal vertical circulation belts. The shallow belt, 0–500 m (Nagy et al., 2019), is the most relevant to the jellyfish in this study. The Atlantic water mass enters through the Strait of Gibraltar and flows generally eastwards via the Strait of Sicily. However, part of the water deviates toward Corsica and exits the MS again, never passing the Strait of Sicily eastwards (Nagy et al., 2019). Most of the water that enters from the Atlantic flows to the eastern MS and then to the Levantine Sea via the Libyan and Israeli coasts. When reaching the Levantine Sea, the water becomes warmer, saltier, denser, and sinks to form the Levantine Intermediate Waters (LIW). The western basin is approximately 3,400 m deep and the eastern basin 4,200 m (Cavaleri et al., 1991; Ignatiades et al., 2009; Malanotte‐Rizzoli et al., 1997; Millot, 1999; Patarnello et al., 2007; Vidussi et al., 2001). The climate is relatively cold in the northern part, with an increasing temperature gradient toward the south (Cavaleri et al., 1991). Therefore, the MS is an ideal model region as it contains many different ecosystems within a relatively small area.

### 1.2 Geology

During the Jurassic and Cretaceous period (208–63 mya) in the Mesozoic era, the Tethys Sea separated Europe from Africa. The exceptional conditions of the MS genesis produced a remarkable diversity in marine life and made it a suitable model of the world's oceans in terms of faunal shifts, invasive species introduction, and spread in marine ecosystems (Hsü et al., 1977; Lejeusne et al., 2010). The eastern MS is a tectonically complex region that has been evolving over a long period of time and is located in the midst of the progressive Afro‐Eurasian collision (Eppelbaum & Katz, 2011). It formed its present shape during the late Miocene (23.03–5.33 mya). Today, the eastern MS is characterized by an arid climate and is considered the most oligotrophic basin in the MS and one of the least productive seas in the world (Azov, 1991).

Around 7.24 mya, a complex combination of tectonic and other processes gradually led to a dramatic geological event that began 5.96 (± 0.02) mya, known as the "Miocene Desiccation of the Mediterranean" or "Messinian Salinity Crisis" (MSC) (Hsü et al., 1973; Krijgsman et al., 1999). This created the Earth's most recent saline giant (an extensive salt deposit, produced by the evaporation of a hypersaline sea). The MSC began with a gradual restriction of seawater exchange between the Atlantic Ocean into the MS. Apparently, a full isolation between those two bodies of water was established between 5.59 and 5.33 mya. The MSC ended at 5.33 mya as the Strait of Gibraltar opened again and new water from the Atlantic Ocean refilled the MS (Grothe et al., 2020; Hsü et al., 1973; Krijgsman et al., 1999; Roveri et al., 2014; Simon et al., 2017). During the MSC, the MS water level reached a minimum of 800–1200 m below the Atlantic sea level (Mas et al., 2018). The low sea level led to high ambient temperatures that accelerated the desiccation process. The exact amount of sea‐level change during this extreme event is still under debate; it is possible that the sea level fell much lower than the suggested 1,200 m (Hsü et al., 1973; Krijgsman et al., 1999; Sternai et al., 2017). This model was supported by the erosion of the sea bed and canyons cut into the slightly older marine sediment (Hsü et al., 1973). Apparently, the MS transformed from a small ocean into a large evaporated pool and then into a brackish water lake—all within 700,000 years (Roveri et al., 2014). Tectonic processes and sea‐level changes triggered the restriction and isolation of the MS from the Atlantic Ocean and the Red Sea, which is pivotal to understanding the oceanographic history of the MS. The MSC had a dramatic effect on Mediterranean fauna. Thus, this remarkable event should be expected to leave some DNA fingerprints on the living creatures of the Mediterranean.

Around 5.33 mya, the MS experienced a new revival, starting with flooding of the desiccated MS through the Strait of Gibraltar. This was the end of the Messinian Salinity Crisis and has been described as the largest known flooding in Earth's history (Garcia‐Castellanos et al., 2009). It is known as the "Zanclean flood” since it started at the beginning of the Zanclean age. A gigantic waterfall characterized this flood, eroding its way down to the desiccated Mediterranean salty lakes (Clauzon, 1973; Garcia‐Castellanos et al., 2009; Popescu et al., 2017). As the MS refilled with water from the Atlantic, the flood may have begun with a smaller discharge that continued for several thousand years. However, Garcia‐Castellanos et al. (2009) suggested that 90% of the water was transferred over a few months, up to a 2‐year time period. Regardless of the time frame, the result of the Zanclean flood was that the Atlantic Sea reclaimed the MS with its fauna and flora. In addition to reaching sea level, this event fostered a new water composition within the MS basin, as the highly salty Mediterranean water blended with fresher Atlantic water, and introduced new Atlantic species to the local, more isolated Mediterranean biota. The removal of the land barrier and the incoming flood introduced Atlantic species, but whether the Atlantic and Mediterranean populations mingled remains controversial (Patarnello et al.,^2007^).

The modern MS marine environment is suffering from major anthropogenic impacts that disturb the ecosystem, such as transportation, climate change, overfishing, degradation of water quality, pollution, and heavy tourism; it makes up 33% of the world's tourism industry (Curr et al., 2000; Jackson et al., 2001). The eastern MS is the world's most affected anthropogenically and zoogeographically marine environment (Golani, 1998), and it is under significant invasion from alien species (Streftaris & Zenetos, 2006). This started with the opening of a waterway, the Suez Canal, between the Indo‐Pacific and the MS in 1869 (Por, 1971). The connection between the two seas caused many marine species to migrate in both directions, either by active or passive transport, or via human transportation. This migration, known as "Lessepsian migration" or "Erythrean invasion," mostly occurred and is still occurring along a migration vector from the Red Sea to the MS and presents a heavy impact on the marine ecosystem (Golani, 1998; Mizrahi et al., 2015). The Suez Canal was widened in 2015 to increase transportation capabilities, and the results from this construction are yet unknown. In the MS, the Suez Canal is likely the main route for introducing nonindigenous species; as much as 53% from all the species in the MS are considered Lessepsian migrants (Galil et al., 2014, 2015). The Aswan High Dam, constructed in 1964, also placed a heavy ecological toll on the marine ecosystem (Golani, 1998). The major effect is the obstruction of sediments (60–180 million tons each year; Sharaf El Din, 1977) and nutrient transportation from the Nile River to the MS as a result (Biswas & Tortajada, 2012; Shalash, 1982).

The marine environment seems to have no visible geographical (allopatric condition) border, as is generally the case in terrestrial environments regarding speciation (Hickerson et al., 2010), but the marine fauna exhibits adaptation and speciation to different ecological or microecological conditions. The oceanographic history of the marine habitat offers changing parameters for the genetic structure of marine species (Neethling et al., 2008; Stopar et al., 2010). Phylogeography is viewing molecular evolution as spatial distribution of genetic lineages (Avise et al., 1987) and is a highly integrative approach used to investigate the relationship between the history of the Earth, ecology, and biotic diversification (Arbogast, 2001;  2009 Pelc et al., 2009; Via, 2009). The dispersal ability of a marine species is related to its phylogeographic capabilities; species with planktonic dispersion abilities possibly reflect contemporary oceanographic changes, while species with restricted dispersal abilities are more likely to reflect historical processes (Galindo et al., 2006; Pelc et al., 2009; Ramšak et al., 2012). Thus, the phylogeographic data we retrieve from marine jellyfish can reveal the geological history of the sea and oceanographic barriers (both visible and invisible), such as currents, temperature, salinity, predation pressure, nutrients. Phylogeographic data also depend on the species’ dispersal ability, and their tolerance to overcome those barriers and become cosmopolitan (Dawson, 2005; Kuo & Avise, 2005; Ramšak et al., 2012).

### 1.3 DNA markers

DNA markers are an important and valuable tool for evaluating taxonomic biodiversity and ecological studies (Hebert, Cywinska, et al., 2003; Hebert, Ratnasingham, et al., 2003). DNA barcoding is rapidly becoming the main approach for identification of species by using genetic markers mostly from mitochondrial DNA (mtDNA) and ribosomal RNA (rRNA). mtDNA is used for a wide range of genetic applications, such as global bioidentification systems, taxonomy, population genetics, and phylogeographical analyses (Avise et al., 1987; Hebert, Cywinska, et al., 2003; Hebert, Ratnasingham, et al., 2003). For example, cytochrome c oxidase subunit I (COI) is a mtDNA gene, used widely as a barcode for species identification and discrimination (Radulovici et al., 2010; Sun et al., 2012). mtDNA is mostly maternally inherited DNA in the zygote, and a lack of heterologous recombination in mtDNA leads to a high probability of its formation from strictly maternal transmission (Hebert, Cywinska, et al., 2003; Hebert, Ratnasingham, et al., 2003; Radulovici et al., 2010; Shearer et al., 2002; Wolff & Gemmell, 2008). Heterologous recombination is a mechanism that can create a distinct class of genomic rearrangements (León‐Ortiz et al., 2018). The ribosomal biogenesis in eukaryotes is taking place in the cytoplasm and in the nucleolus. The rRNA gene, also known as ribosomal DNA (rDNA), is a tandem repetitive cluster and is the most abundant gene in the eukaryotic genome. Since it is required for the ribosome biogenesis, rDNA is critical for the cell's functions and is highly conserved from bacteria to humans (Dentinger et al., 2011; Kobayashi, 2008; Takehiko Kobayashi, 2011; Lam et al., 2007; Schoch et al., 2012). By combining multiple genes, as the nuclear 18S rDNA and 28S rDNA genes, it is possible to attain longer nucleotide data in order to reconstruct the evolutionary impact on a species (Li et al., 2012).

In this work, we used the most appropriate genetic tool for our research question (locating the small changes in base pairs of the genetic code for a species) and recent innovations in molecular technologies (Komoroske et al., 2017). Most marine jellyfish genetic studies to date have used mtDNA. Nevertheless, our work is dependent on worldwide data available for comparison with our collected data. This data limitation led us ultimately to using the more classic genes like COI, 18S and 28S. To create a reliable evolutionary clock tree (timetree) and determine the molecular clock for our examined scyphozoa, we found the most appropriate gene for sequence alignment and a proper outgroup taxon. We calibrated the specific taxon divergence time, which is the basis for calculating nucleotide rates of change and for forming a timetree (Hedges et al., 2006). The DNA mutation rate is unique for each taxon; here, we tracked the most recent common ancestor to establish our nearest taxon nucleotide rate of change. With this database, we calculated the divergence time for our specific jellyfish and calibrated the molecular clock. Scyphozoa have a life cycle with a pelagic predatory stage involving complex senses and traits (Cartwright et al., 2007). Although there are many fossils of medusoid‐like animals from the late Neoproterozoic and Cambrian period, most of them lack distinctive evidence of the soft‐part anatomy (Cartwright & Collins, 2007; Cartwright et al., 2007). Recent methods utilizing the geological and genomic records enable us to estimate divergence times based on the limited number of fossil records (Hedges & Kumar, 2003; Park et al., 2012; Peterson et al., 2007) and made it possible to calibrate the phylogenetic evolutionary timetree nodes for our examined scyphozoa (Cartwright & Collins, 2007; Nowak et al., 2013; Ronquist et al., 2012). Usually, the classic molecular clock refers to the evolutionary rate as constant (Zuckerkandl & Pauling, 1962). However, there is much variability in mutation rates between taxonomic groups, and these are affected by population size, generation length, and other factors. To minimize our mtDNA mutation rate error, we adopted the local clock based on a publication from Yoder and Yang (2000).

Jellyfish possess some unique traits that make them suitable as genetic biological indicators for aquatic environments. Their body is constructed from gelatinous soft tissue with a water content of 95% (Hsieh et al., 2001; Tucker, 2010), and they do not have any solid skeleton or shell. Thus, they are highly exposed to the physics and chemistry of the water column. These gelatinous zooplankton are large, exhibit slow swimming speeds, and lack highly complex behavior (Hamner & Dawson, 2009; Larson, 1992). They are highly dependent on environmental conditions and on their flexibility to adapt. They cannot escape, swim, or migrate to a new environment like fish or other creatures with more complex behaviors. Jellyfish are among the oldest creatures to inhabit the Earth and are among the first multicellular creatures that left their sessile state and began swimming and wandering in the oceans (Fedonkin & Waggoner, 1997). Fossil evidence indicates that the cnidarians originated in the early Cambrian, and some of today's jellyfish species resemble their ancestors' body shape (Cartwright & Collins, 2007; Cartwright et al., 2007; Han et al., 2010).

We selected three representative scyphozoa from among the Mediterranean jellyfish species that represent different means, vectors, strategies of migration, and phylogeographic patterns (Mizrahi, 2014; Mizrahi et al., 2015) in order to achieve a more detailed picture of the processes occurring in the MS. These three jellyfish species are meroplanktonic and are spending the greater parts of their lives in the benthic region. Their reproduction is characterized by the alternation between sexual and asexual states.

### 1.4 Rhizostoma pulmo

**FIGURE 1:**
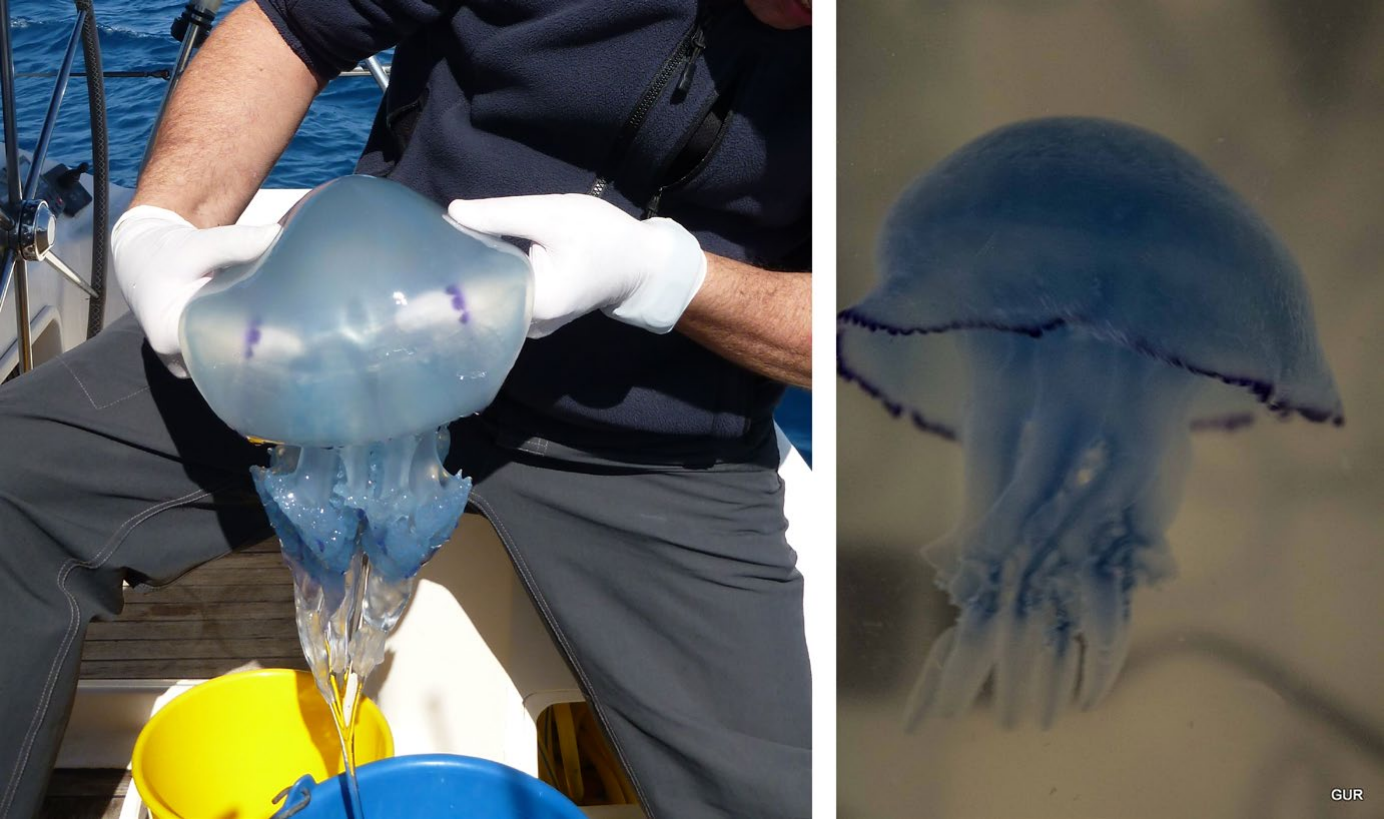
*Rhizostoma pulmo* in Haifa Bay, Israel: collecting *R. pulmo* of different sizes, in winter and summer seasons. On the left, a specimen from 20 February 2013, about 35 cm in length. On the right, a specimen from 8 July 2013, about 15 cm in length

**FIGURE 2:**
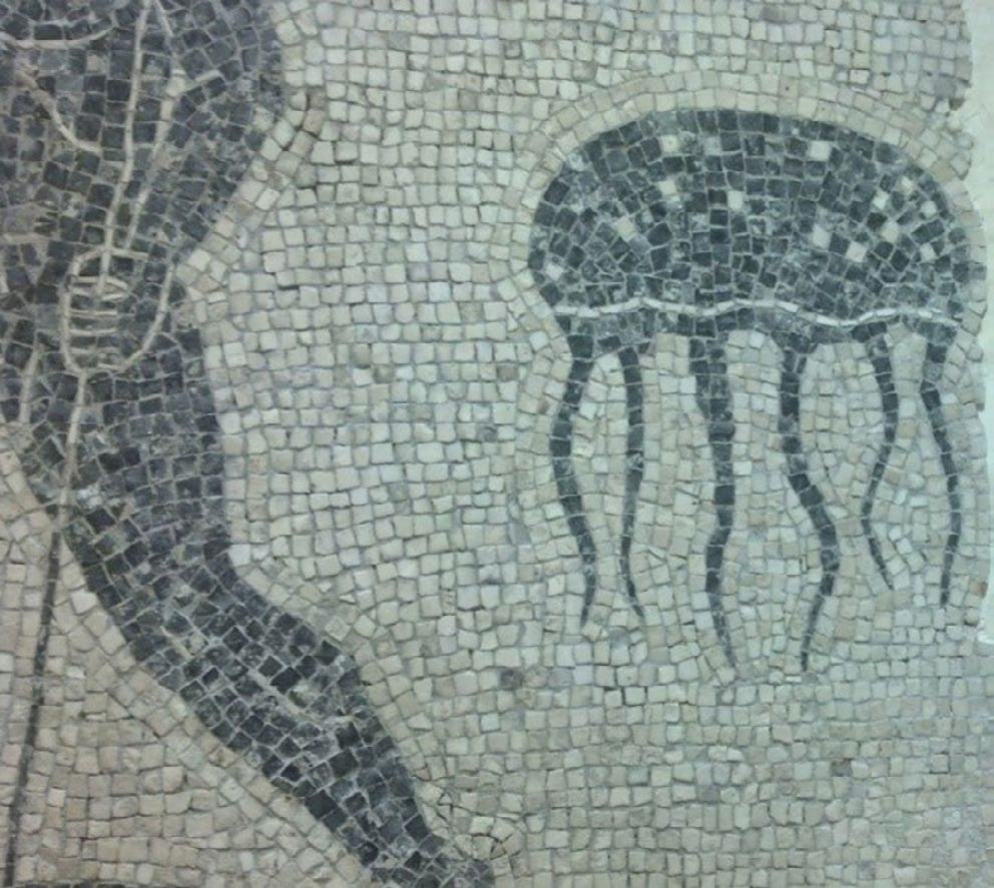
Jellyfish depicted in an ancient mosaic from the 5th century, Jerusalem, which potentially indicates that jellyfish were known in ancient times in this area. The depicted jellyfish is either *Rhizostoma pulmo* or *R*. *nomadica*. Source: Dr Beverly Goodman, University of Haifa

*Rhizostoma pulmo* (Macri, 1778) is also known as the barrel jellyfish. *R*. *pulmo* is native to the northern Atlantic, was first documented in 1875, and has been frequently observed since, except for the period between the years 1930 and 1960 (Kogovšek et al., 2010). In the Levant Basin, *R*. *pulmo* (Figure 1) is known as the "Common Jellyfish" and was first reported along the Israeli coast by Bodenheimer (1935). Israeli *R*. *pulmo* specimens have been collected and recorded continually since 1990 (Galil et al., 1990; Mizrahi, 2007). One of the oldest known images of a jellyfish from the region is a mosaic from the 5th century A.D., in Jerusalem. The image resembles *R*. *pulmo* or *Rhopilema nomadica* (Figure 2), the species that are most abundant in the region today. From the Lebanese Mediterranean waters, there are reports since 1971 (Galil et al., 1990) with records of *R*. *pulmo* proliferating during 1986 (Turan & Ozturk, 2011). In northern Cyprus, the *R*. *pulmo* species reports have stated that abundance is rare and it has been recorded mostly during the months March to August (Turan & Ozturk, 2011). Historically, *R*. *pulmo* blooms in the northern Adriatic Sea were first recorded from 1883 and cited by Avian and Rottini Sandrini (1994), and have been very common in this region (Kogovšek et al., 2010). There is evidence that *R*. *pulmo* blooms in European seas were confined to semi‐enclosed bays that received distinctly more fresh water and nutrients from rivers. This may be the reason for the more frequent observations of *R*. *pulmo* along the western coast of the North Adriatic, where influence from fresh water is more pronounced (Kogovšek et al., 2010; Lilley et al., 2009). Bosch‐Belmar et al. (2017) claim that *R*. *pulmo* is among the most sighted species in the following countries: Italy, Spain, Tunisia, and Malta. From the journal *Taxonomy* (2017), there are 6,142 jellyfish observation reports of *R*. *pulmo* from the year 1898 until 2017, with data collated from United Kingdom, Denmark, Italy, France, Germany, Bulgaria, Georgia, Spain, and the Netherlands. From the North Atlantic Ocean, we have data of *R*. *pulmo* abundance (Costello, 2001), but some data from the South Atlantic as well (Horton et al., 2018). Furthermore, a lone report exists from Pakistani waters (Muhammed & Sultana, 2008). Wavelet analysis showed that the periodicity of occurrences has shortened in recent decades and the recurrence of blooms has increased (Kogovšek et al., 2010). We can consider *R*. *pulmo* as the most representative scyphozoa in the MS, as it is well documented throughout the Mediterranean and adjacent seas. In a published phylogeographic study on *R*. *pulmo* from 2012 (Ramšak et al., 2012), the authors concluded that "no genetic structure was detected in *R*. *pulmo* from the MS," but the extensive presence of *R*. *pulmo* jellyfish throughout the Mediterranean requires us to find out if that is really the case.

### 1.5 *Aurelia* sp

*Aurelia aurita* (Linnaeus, 1758) is known as the moon jellyfish and is the most‐studied group within gelatinous zooplankton. It is probably the most displayed Scyphozoa in aquaria. Although this genus is well studied, its taxonomic status remains unclear due to its high morphological variability (Schroth et al., 2002). The number of *Aurelia* species is controversial among scientists and was changed frequently over the years. Part of this uncertainty emanates from traditional taxonomic methods (Häussermann et al., 2009). Though Mayer (1910) suggested there are 12 species belonging to the genus *Aurelia*, Häussermann et al. (2009) stated that by the end of the 20th century only two species were recognized. Kramp (1961) describes seven species and in a later work, Kramp (1968) describes as much as 20 *Aurelia* species. However, DNA analyses revealed 16 phylogenetic species (Schroth et al., 2002), and today, the number of 13 known species of *Aurelia* is commonly accepted, although this number is not final. This confusion only emphasizes the need for molecular tools to assist classification. Dawson (2003) stated "Molecular data therefore provide an important opportunity to evaluate independently the utility of morphological data in systematic studies." Dawson and Martin (2001) pointed out that, despite morphological complications concerning the *Aurelia* genus, there were molecular analyses employed that identified the same divisions as did morphological analysis.

**FIGURE 3:**
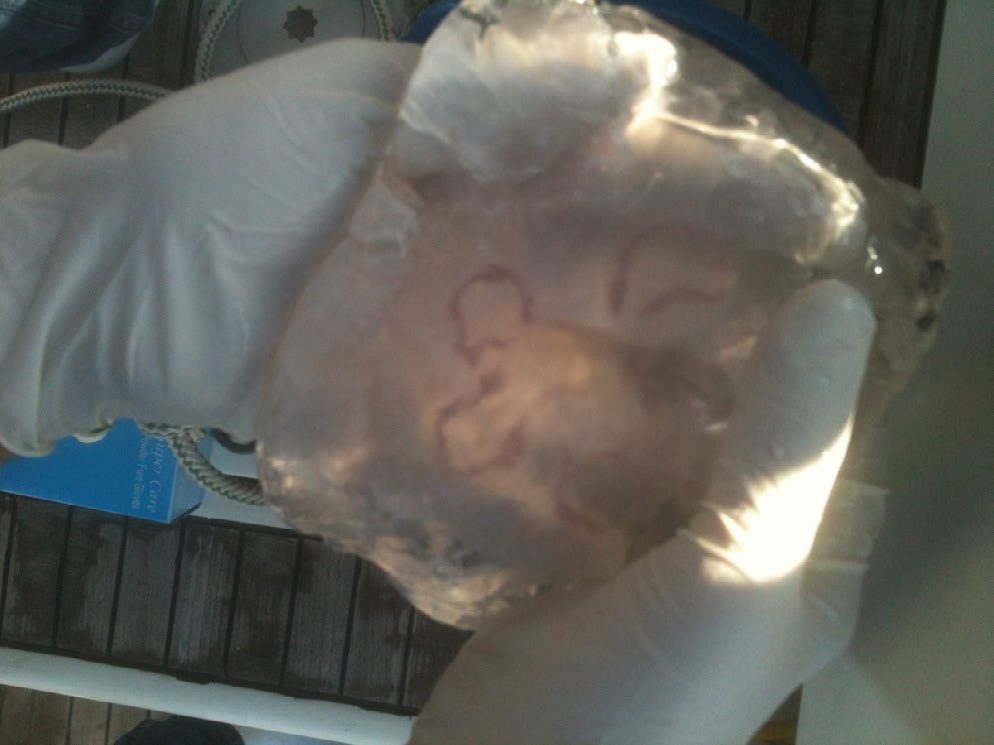
An eastern Mediterranean *Aurelia* sp. specimen in Haifa Bay, Israel, and its horseshoe‐shaped gonads

*Aurelia* sp. is considered a cosmopolitan species with a worldwide distribution in neritic waters between 70°N and 55°S (Dawson & Martin, 2001). They can be found in different coastal and continental shelf marine environments and have been reported in Japan, North America, the Black Sea, northwestern Europe (Lucas, 2001), and in semi‐enclosed bays and inlets (Purcell et al., 2000). In the Israeli eastern MS, *Aurelia aurita* has been observed for a long time, but recently their abundance has decreased. The first record of *Aurelia aurita* was in 1935 by Bodenheimer (1935). In Israel, samples were collected and reported at Beit Yanai in 1972, 1983, and 1984 (Galil et al., 1990). As for our work, finding specimens of *Aurelia aurita* in the MS (Figure 3) is of special interest because this jellyfish is very common in the Israeli Red Sea at Eilat, on the northern shores of the Red Sea in the Gulf of Aqaba. The *Aurelia* sp. is an example of a scyphozoa existing in both seas and can describe a potential relationship between the two populations of the *Aurelia* sp. *Aurelia* sp. can serve as a good model to demonstrate the power of organismal, ecological, and molecular data, as suggested Schroth et al. (2002). Phylogeographic analyses were conducted worldwide on *Aurelia* sp. by using DNA markers and revealed cryptic species, demonstrating that *Aurelia* sp. is distinctly separated from other *Aurelia* species and exhibits comparative or parallel phylogeographic patterns (Dawson & Jacobs, 2001; Ramšak et al., 2012). Ramšak et al. (2012) concluded that the *Aurelia* sp. is successfully distributed in the MS.

### 1.6 Phyllorhiza punctata

**FIGURE 4:**
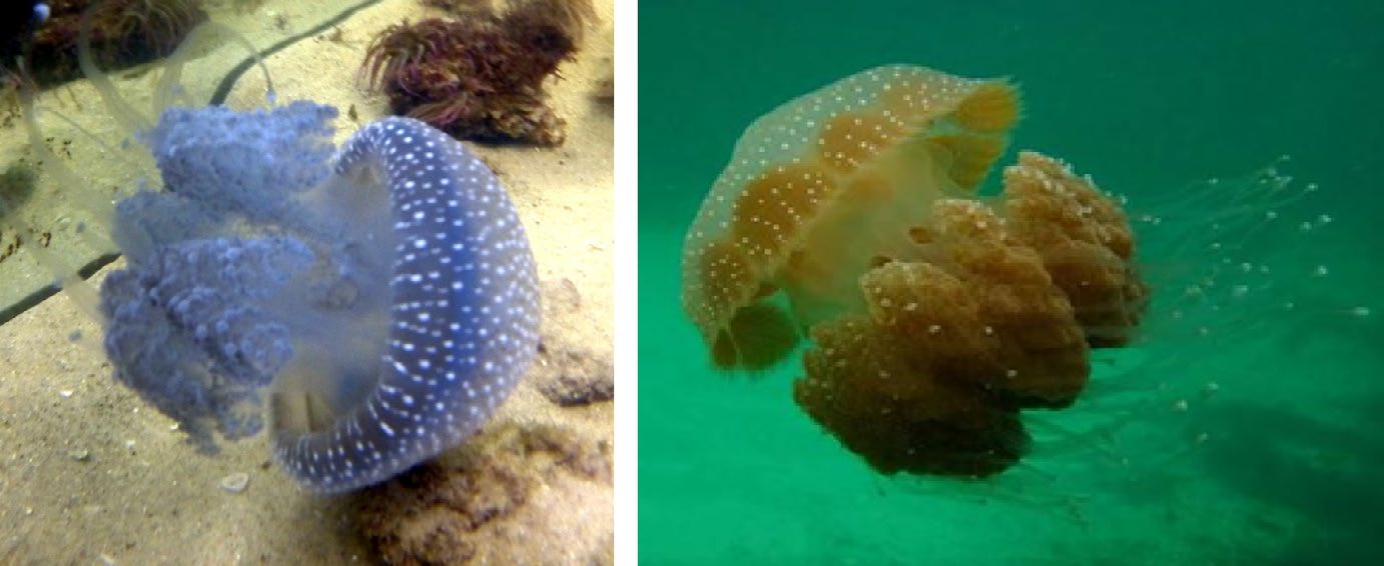
*Phyllorhiza punctata* off the coast of Acre, Israel (L). In Australia, source: Adam Greenberg (R)

*Phyllorhiza punctata* (von Lendenfeld, 1884; Figure 4) was first recorded outside of the Indo‐Pacific Ocean only after the mid‐20th century and was likely introduced to the MS by means of vessel transportation (Galil et al., 2009). The first record and reported observation in the MS was from the Israeli shoreline in 1965 at Beit Yanai (Galil et al., 1990). Since then, it has been reported in different Mediterranean locations (Abed‐Navandi & Kikinger, 2007; Boero et al., 2009; Galil et al., ,^1990^, ^2009^; Gülşahin & Tarkan, 2012).

**FIGURE 5:**
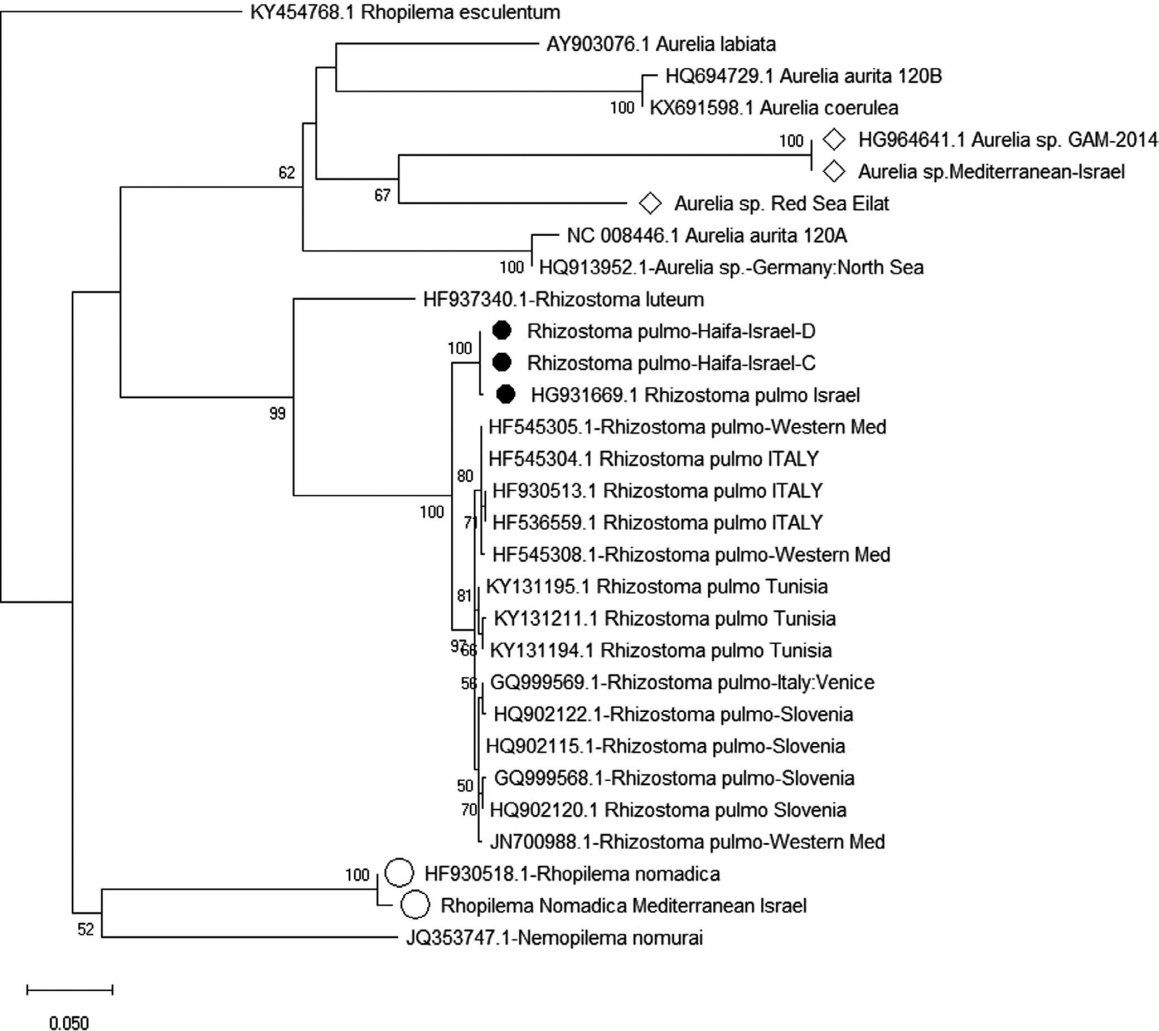
Evolutionary tree calculated by using 508 bp of partial COI mtDNA markers. The full black circle marks the Levantine *Rhizostoma pulmo* samples, the empty rhombus represents the Levantine *Aurelia* sp., and the empty circle represents the Levantine *Rhopilema nomadica*

*Phyllorhiza punctata* and *Rhopilema nomadica* (Figure 5) are two jellyfish species that are considered Lessepsian migrants and new immigrants to the Mediterranean (Galil et al., 1990). *P*. *punctata* (Figure 4) is indigenous to the tropical Western Pacific and is mainly distributed in Australian, Philippine, and Japanese waters (Graham et al., 2003; Mariottini & Pane, 2010). In the years 2005 and 2006, impressive numbers of *P*. *punctata* were reported after many years, wherein it was very rare or not sighted at all in the Levant (Mizrahi, 2014). In the 1950s, *P*. *punctata* was recorded in southern Brazil and was misidentified and considered a new species until the misconception was detected. With the help of genetic tools, we aim to determine whether the presence of *P*. *punctata* in the MS is new and caused by human interference and to understand its migration vector. The abundance of *P*. *punctata* is fluctuating, like most jellyfish populations. In 2013, it was highly abundant in the eastern MS (Mizrahi, 2014).

## 2 MATERIALS AND METHODS

DNA markers from three thriving Mediterranean macro‐jellyfish species were used for aligning the sequences. This genetic data analysis is based on our own collection of samples and on supplementary existing data from GenBank (Geer et al., 2009).

### 2.1 Sample collection

Between 2012–2017, the jellyfish were collected in the northern part of the Mediterranean Israeli shoreline—32°33′N, 034°50′E to 33°00′N, 035°05′E—including Haifa Bay (Figures 1 and 3) and the shoreline of the Red Sea in Eilat—29°30′N, 034°55′E to 29°32′N, 034°58′E. In total, we collected 126 jellyfish specimens from the three species.

### 2.2 DNA amplification and sequencing

Samples from the collected jellyfish were immediately processed in situ (Figures 1 and 3). Sample cuts from the oral arms or gonads were excised and, for most of them, DNA extraction was initiated in situ. Total genomic DNA was extracted using the Wizard® SV Genomic DNA Purification System kit (Promega). Other samples were preserved in situ in 95% ethanol for further processing in the laboratory. Throughout the work, care was taken to use sterilized tools and containers, and gloves were worn. The genetic classification was done by aligning DNA sequences to global and local data to achieve the most accurate species identification for the sampled specimens (Bayha et al., 2010; Pett et al., 2011). We used "universal" DNA primers (Folmer et al., 1994) as well as our own primers that were developed by our laboratory (Table 1). DNA concentration was measured using NanoDrop, absorption at 260 nm. DNA preparation was done using the Promega kit for polymerase chain reaction (PCR) and sequencing amplification. After a final dilution to 2 ng/µl, DNA quality was assessed by running samples on 1% agarose gels. All amplifications were carried out in a T100™ Thermal Cycler (BIO‐RAD) using GoTaq® Green Master Mix (Promega). PCR products were purified using the Wizard® SV Gel and PCR Clean‐Up System (Promega). Sanger sequencing of the specimens was performed by "HyLabs" Israel. DNA sequences were partly submitted (including corresponding primers) as in Table 1 to the European Nucleotide Archive (ENA).

**TABLE 1:** List of Rhizostomatidae nucleotide gene primers database

Primers for: Eukaryota, Metazoa, Cnidaria, Scyphozoa, Rhizostomeae, Rhizostomatidae		
Gene primer for: Primer forward name:	16S rDNA F756	CCG TGA TAA AGT AGC ATA ATCA C
Gene primer for: Primer reverse name:	16S rDNA R755	AAT ATT ACC CTG TTA TCC CTA CGG
Gene primer for: Primer forward name:	28S rDNA F834	GAG ACC GAT AGC GAA CAA GTA CCG TG
Gene primer for: Primer reverse name:	28S rDNA R833	AGA GTT TCC TCT GGC TTC ACC CTA CTC
Gene primer for: Primer forward name:	COI mtDNA F314	GAA CTA TCA GGG ACG GGA TCT AT
Gene primer for: Primer reverse name:	COI mtDNA R313	GTG GAA ATG AGC AAC AAC GTA AT
Gene primer for: Primer forward name:	18S rDNA F315	GGT ATG TTA CTG GCT GGT CTG
Gene primer for: Primer reverse name:	18S rDNA R316	ACC TTG TTA CGA CTT TTA CTT CCT C

Notes

All nucleotide gene primers were uploaded to the GenBank with respect to their DNA sequences.

### 2.3 Evolution tree reconstruction

Sequences were manually trimmed, edited, and aligned using BioEdit version 7.2.5 (Hall, 1999). Evolutionary trees (Figures 5, 7 and 8) were created using MEGA X (Kumar et al., 2018). To test what is the best fit for evolution models we used MEGA X “Find Best DNA/Protein Models Maximum Likelihood (ML)” (Kumar et al., 2018). Evolutionary history was inferred by using the maximum likelihood method based on the general time‐reversible model (Nei & Kumar, 2000), using neighbor‐joining analysis (Mega X). The initial tree for the heuristic search was automatically obtained by applying Neighbor‐Join and BioNJ algorithms to a matrix of pairwise distances and then was estimated using the maximum composite likelihood approach, and then the topology (with the superior log likelihood value) was selected. Specific parameters for each analysis are listed below.

### 2.4 *Rhizostoma pulmo* (Figure 5)

The evolutionary tree (Figure 5) focuses on the MS *R*. *pulmo* population. The outgroup is *Rhopilema esculentum*. Maximum Likelihood of Evolutionary Trees for *R*. *pulmo* was calculated by using 508 base pairs (bp) of partial COI mtDNA marker using model = GTR + I, Ts/Tv = 4.6677, Invariant = 0.5972, with 1,000 bootstrap replications. Number of Threads = 3, No. of Seqs: 30, SBL = 1.91694614, Log Likelihood = −2904.77 (Kumar et al., 2018).

### 2.5 *Aurelia* sp. (Figure 7)

This evolutionary tree (Figure 7) focuses on the worldwide dispersal of *Aurelia* sp. population with an emphasis on the eastern MS in Haifa Bay and the Israeli Red Sea (Eilat) *Aurelia* sp. There was a total of 658 bp of partial COI mtDNA marker used in the final dataset. The evolutionary history was constructed using the maximum likelihood method based on the general time‐reversible model (Nei & Kumar, 2000). The tree with the highest log likelihood (−5091.54) is shown. A discrete Gamma distribution was used to model evolutionary rate differences among sites (five categories (+G, parameter = 4.2722)). The rate variation model allowed for some sites to be evolutionarily invariable ([+I], 59.89% sites). The tree is drawn to scale, with branch lengths measured for the number of substitutions per site. The analysis involved 43 nucleotide sequences. Codon positions included were 1st + 2nd + 3rd + Noncoding. Evolutionary analyses were conducted in MEGA X (Kumar et al., 2018). The number of bootstrap replications is 1,000.

### 2.6 *Phyllorhiza punctata* (Figure 8)

This evolutionary tree shows the worldwide dispersal of *Phyllorhiza punctata*, built on 18S and 28S rDNA markers. The evolutionary tree of *P*. *punctata* (Figure 8) is composed of two combined rDNA markers, 18S and 28S. As a result of a lack of global genetic information, and in order to achieve longer alignment sequences, we used the technique of connecting two different segments of genes that overlap and then manually trimmed them. There was a total of 2,844 bp positions of 18S and 28S rDNA markers, (or nucleotide pairing) in the final dataset. The evolutionary history was constructed by using the maximum likelihood method based on the model of Kimura 2‐parameter (Kimura, 1980). The tree with the highest log likelihood (−5197.16) is shown. The analysis involved seven nucleotide sequences. Codon positions included were 1st + 2nd + 3rd + Noncoding evolutionary analyses were conducted MEGA X (Kumar et al., 2018). The number of bootstrap replications numbered 1,000.

### 2.7 Timetree (Figure 6)

**FIGURE 6:**
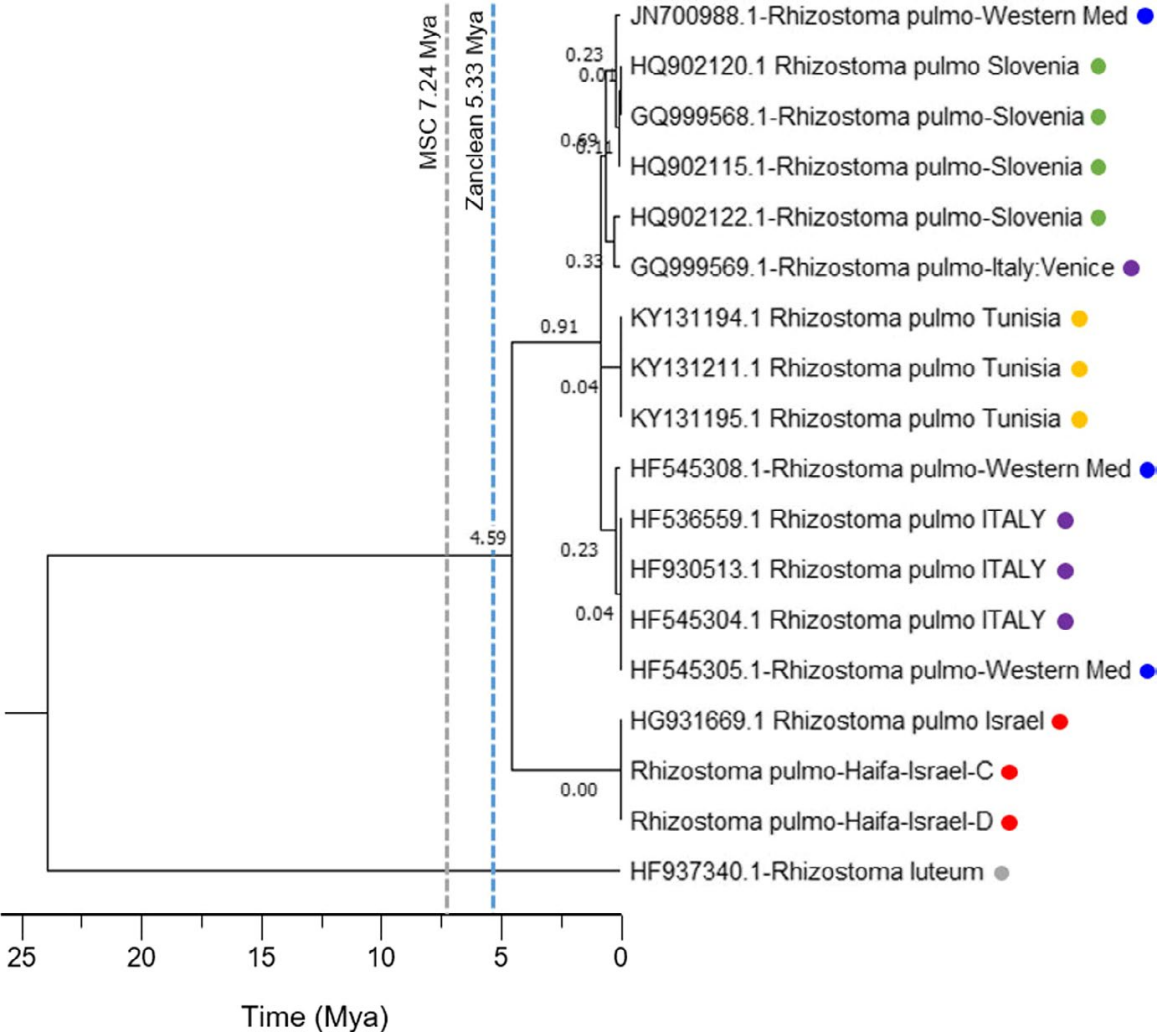
Evolutionary timetree of *Rhizostoma* *pulmo*. The timetree shows the divergence time in a scale of mya. Notice the divergence time of 4.59 million years ago (mya), which separates Israeli *R*. *pulmo* samples from the rest. The "Messinian Salinity Crisis" (MSC, dashed gray) and the Zanclean flood (dashed light blue) times estimates are marked. Color represents *R*. *pulmo* sampling locations: red—Israel; purple—Italy; blue—western MS; yellow—Tunisia; green—Slovenia. *Rhizostoma luteum* (gray) was used as an outgroup

This timetree focuses on *Rhizostoma pulmo* population (Figure 6). Sequences were manually trimmed, edited, and aligned using BioEdit version 7.2.5 (Hall, 1999). The evolutionary tree and molecular clocking were conducted using MEGA X (Kumar et al., 2018). To find the best fit for the popular evolution models, we used the MEGA X “Find Best DNA/Protein Models Maximum Likelihood (ML)” (Kumar et al., 2018). We computed (Figure 6) an evolutionary clock tree (also known as an evolutionary timetree) and determined the molecular clock to understand the Mediterranean oceanographic processes and historical biogeography with the help of DNA markers. To achieve this objective, we needed to estimate rate and time of divergence of the jellyfish species (Peterson & Butterfield, 2005; Thorne & Kishino, 2002). Our model is based on an estimated relative node for the divergence time from the most recent common ancestor (MRCA) in our taxonomic scyphozoan groups (Thorne & Kishino, 2002).

### 2.8 Rate of change calibration

In order to calibrate the divergence time and rate of change, we used existing published data describing the history of three recent, common ancestral scyphozoa (Geer et al., 2009; Park et al., 2012). We employed two divergence points to achieve one calibration constraint time rate. The first divergence time node we used was a point between two members of Scyphozoan class *Chrysaora quinquecirrha*, (accession ID: HQ694730) and *Aurelia* sp. (accession ID: NC_008446 and HQ694729) whose divergence occurred around 440 mya, apparently at the beginning of the Silurian period. The second divergence point was based on two species of *Aurelia* sp. (accession ID: NC_008446 and HQ694729), whose divergence occurred around 120 mya, in the Cretaceous Period (Park et al., 2012). This allows us to calibrate the rate of mtDNA changes, and both minimum and maximum divergence time enabled us to calibrate the DNA evolutionary clock and determine the mtDNA rate of change for the examined jellyfish.

### 2.9 Timetree construction

A timetree (Figure 6) was constructed using a real‐time method (Kumar et al., 2018) and the Tamura‐Nei model (Tamura, 1992). The timetree was computed using 508 bp of the partial COI mtDNA marker and one calibration constraint. The estimated log likelihood value is −2478.00. The analysis involved 26 nucleotide sequences. The 26 nucleotide sequences included 18 representatives of *Rhizostoma* sp. and eight representatives of *Aurelia* sp., as shown in Figure 5. Figure 6 is scaled to show the *Rhizostoma* sp. population more clearly and we presented 17 individuals of *Rhizostoma pulmo* and one *Rhizostoma* *luteum* as our outgroup in the presented timetree (Figure 6). Codon positions included were 1st + 2nd + 3rd + Noncoding. Evolutionary analyses were conducted in MEGA X (Kumar et al., 2018).

## 3 RESULTS

### 3.1 *Rhizostoma pulmo* DNA reveals existing East and West MS populations

To assess the phylogeography of *Rhizostoma pulmo*, we analyzed the available published data of *R*. *pulmo* (Geer et al., 2009) and our newly collected *R*. *pulmo* specimens from the Levant (marked with a full black circle, Figure 5). In addition to *R*. *pulmo* specimens in the tree (Figure 5), we presented other groups of jellyfish commonly present in the Mediterranean that are highly similar in size and shape and usually appear together with *R*. *pulmo* (Figure 5). Those include *Rhizostoma luteum*, *Rhopilema nomadica*, *Nemopilema nomurai*, four different *Aurelia* species, and *Rhopilema esculentum* as an outgroup. This tree (Figure 5) displayed a wider picture of those jellyfish that inhabit the Mediterranean Sea and its neighboring seas. The tree focuses on the MS *R*. *pulmo* population. Results emphasize the difference in a distinct way between the eastern and western MS *R*. *pulmo* populations. Although in the western part of the MS we see a separation of the groups, it is not as clear as the difference between the eastern and western MS.

### 3.2 *Rhizostoma pulmo* timetree reveals the divergence time of East and West MS populations

The timetree (clock tree) for the Mediterranean *R*. *pulmo* was created by using the DNA marker of mtDNA cytochrome c oxidase subunit I (COI). The graph (tree) shows a clear picture of the divergence of *R*. *pulmo* into different populations (Figure 6) and in agreement with the phylogenetic analysis (Figure 5). The most distinct point in the timetree is the divergence that occurred around 4.59 mya and gives us a picture of the separation between the eastern and western *R*. *pulmo* populations at this time point. In this tree (Figure 6), the emergence of subgroups that are happening at a much later time can be seen, and this change mostly points to a process that likely occurred and is still ongoing in modern times (Figure 6).

### 3.3 Evolutionary tree of *Aurelia* sp. shows unique population and separation between Red Sea and MS populations

**FIGURE 7:**
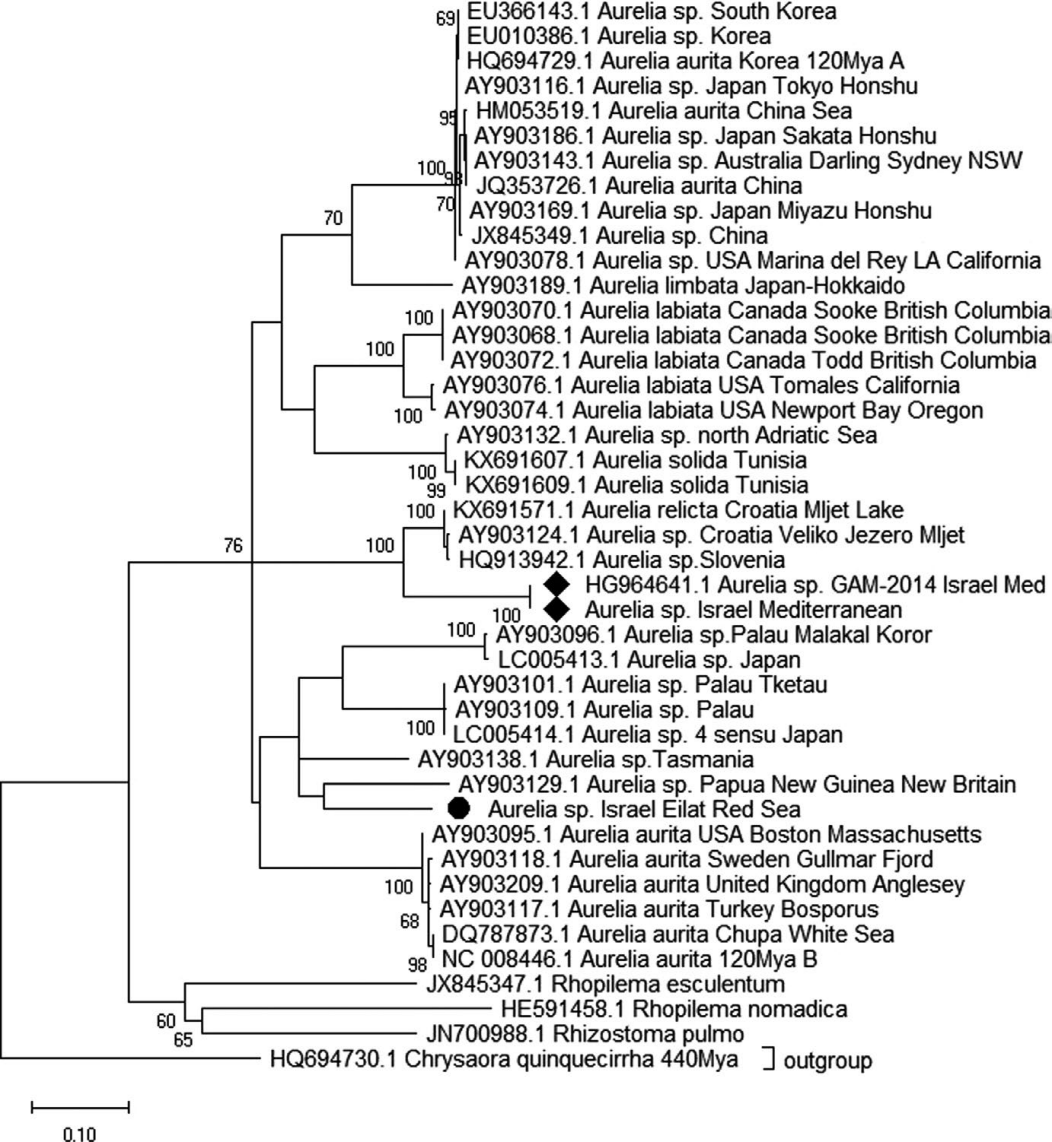
Evolutionary tree focusing on *Aurelia* sp. generated using the COI mtDNA marker. The tree exhibits the worldwide dispersal of *Aurelia* sp., with emphasis on the eastern MS (Haifa Bay, Israel; black rhombus) and the Red Sea (Eilat, Israel; black circle)

Since *Aurelia sp*. is among the few scyphozoan species found both in the MS and the Red Sea, we focused on this species to study connectivity between populations in the two seas and potential migration across the Suez Canal. We constructed an evolutionary tree of *Aurelia* species (Figure 7), using the COI mtDNA marker based on data gathered from around the world and samples collected in this study from the eastern MS (Haifa, Israel) and the Red Sea (Eilat, Israel). The eastern MS *Aurelia* sp., from Haifa Bay (Figure 3), is marked with a black rhombus and the Israeli Red Sea *Aurelia* sp. is indicated by a black circle. This tree allows us to visualize the biological connections between the different *Aurelia* sp. populations that inhabit different areas and allows us to understand the possible migratory vectors, if they existed. The tree emphasizes the distance between eastern MS and Red Sea *Aurelia* specimens, as the latter clustered closer to samples from the Pacific Ocean while the former clustered with samples from Slovenia and Croatia. These results may imply that the populations from the eastern MS and the Red Sea are separated and do not mix.

### 3.4 *Phyllorhiza punctata* DNA reveals immigration trajectories from the Pacific to the MS

**FIGURE 8:**
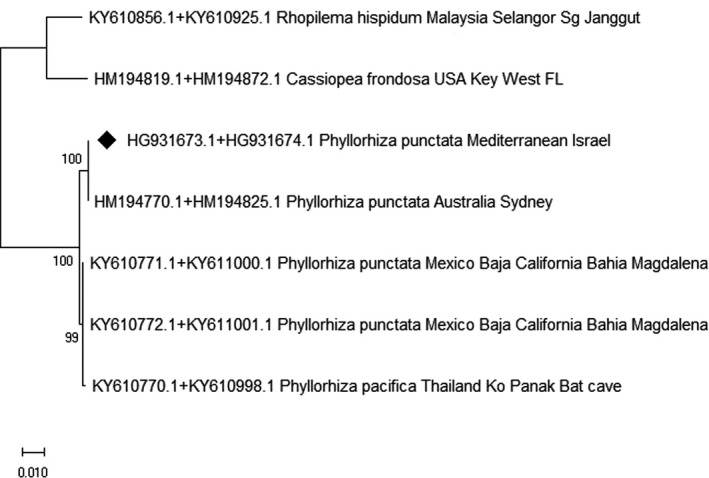
Evolutionary tree built on 18S and 28S rDNA markers for the worldwide dispersal of the *Phyllorhiza punctata*

The evolutionary tree (Figure 8) was built using the 18S and 28S rDNA markers and shows the worldwide dispersal of *P*. *punctata* (Figure 8). The eastern MS *P*. *punctata* is marked with a black rhombus (Figure 8). This tree allows us to view the biological connections between the different *P*. *punctata* populations that inhabit different seas, and thus, we may better understand the possible migratory vectors, if they existed. According to the results, the eastern MS specimens clustered with samples from Australia and then with more distant related samples from Mexico and Thailand. These results indicate recent migration to the MS, apparently by long‐distance transportation (anthropogenic vector), with no data or reports for this species between those seas. Thus, we suggest that *P*. *punctata* populations in the MS likely originate from the Pacific Ocean.

## 4 DISCUSSION

The current outline of the Mediterranean Basin is a consequence of geological history and has a drastic influence on jellyfish biodiversity (Casal‐López & Doadrio, 2018). In this work, we investigated historical geologic events and contemporary processes by taking advantage of evolutionary forces that triggered and promoted genetic changes. We are attempting to understand the correlation and the relationship, if any, among genetic alternations and environmental processes.

### 4.1 *Rhizostoma pulmo* as a valued genetic biological indicator

*R. pulmo* was described in a recent study as a jellyfish species without any trace of genetic structure across the MS (Ramšak et al., 2012). Our DNA results of *R*. *pulmo* (Figures 5 and 6) reflect the opposite and present this jellyfish as an important phylogeographic species. These novel DNA results were made possible by trace evidence obtained from the eastern MS *R*. *pulmo*. The results present a slow and long‐term shift across the MS. *R*. *pulmo* inhabits all parts of the MS, and as such, it is among the most valued biological indicator organisms to monitor MS marine changes.

### 4.2 *Rhizostoma pulmo* reveals a new boundary in the Mediterranean Sea

The results of Figure 5 emphasize the difference between the eastern and western MS *R*. *pulmo* populations in a distinct way. Although we see separation into groups in the western part of the MS, this separation is not as clear as the division between the eastern and the western MS populations. For the first time, we found that the genetic results for the jellyfish we examined do not match the commonly accepted demarcation of the MS to the east and west of the Strait of Sicily and that this demarcation is significant to the east, toward the Levantine Sea. Thus, we grouped jellyfish from Slovenia with the western MS jellyfish because of their DNA results. It is reasonable to suggest that marine barriers for jellyfish are possibly different from marine barriers for other marine animals. Unfortunately, the existing data are not sufficient to draw firm conclusions, and we require more research to collect more DNA data from the MS to find the precise demarcation line between the eastern and western Mediterranean Sea according to jellyfish.

### 4.3 History of the Mediterranean Sea revealed by *Rhizostoma pulmo* DNA timeline

In Figure 5, the Levantine *R*. *pulmo* (marked with a full circle) appears as a separate and isolated branch from the other central and western MS *R*. *pulmo* populations. In Figure 6, the tree presenting the molecular evolutionary clock (timetree) shows when this genetic change (divergence time) happened and formed the separation between the two groups of *R*. *pulmo* (Figure 5). This speciation and dispersal of the eastern *R*. *pulmo* from the central and western populations of *R*. *pulmo* occurred sometime around 4.59 mya (Figure 6). To explain this divergence on the genetic molecular clock, we compared these DNA timetree data with the MS geological history timeline. Based on these data, we synchronized both timelines, of jellyfish and geographic history, and proposed the following timeline to describe the changes over the period of about 6 million years in the MS. Our timeline of the genetic results starts at the end of the Miocene geological epoch. This is when the Strait of Gibraltar became a barrier during the Messinian Salinity Crisis and caused the MS to be partly or fully isolated at around 5.9 mya. It became a brackish water lake or possibly several lakes. The water level was dramatically low and reached a minimum of 800–1,200 m (Mas et al., 2018). The Strait of Gibraltar barrier opened again at around 5.33 mya. This was accompanied by a huge flooding known as the "Zanclean flood." The huge flow of seawater from the Atlantic transferred 90% of the final water volume in a period of a few months to 2 years (Garcia‐Castellanos et al., 2009) and created a new, mixed water composition. The removal of the land barrier and the incoming flood introduced Atlantic species (Patarnello et al., 2007) and also mixed and dispersed those species that survived the long salty period. A new physically and chemically uniform water body was created from the salty MS and allowed *R*. *pulmo* to establish itself all over the MS. Our *R*. *pulmo* ancestors may have successfully and effectively made a habitat in this new water body. Examining the *R*. *pulmo* timetree (Figure 6), it took as much as 740,000 years (from 5.33 to 4.59 mya, Figure 6) for the revived MS to stabilize and to produce some environmental differences between the eastern and western MS areas. During this time, *R*. *pulmo* could interact with other populations and was not hindered by any unseen barrier. As we revisit the historical timeline of this species’ clock to the 4.59 mya time point, we can focus and suggest some scenarios for this exceptional finding.

It may have taken about 740,000 years after the Zanclean flood for the MS to stabilize and adapt to the geographic structure and the physical conditions. But it is more likely that a dramatic environmental event created some sort of invisible marine barrier between the western and eastern MS. This dramatic event could have been a result of certain phenomena, as can be supported by new research pointing at dramatic events in the same time period, 4.59 mya, as our results. An arid period, tectonic activity, high radiation, volcanic eruptions that affected the temperature, and currents of the sea (Sandoval‐Castillo & Beheregaray, 2020) are some of the possibilities. Another study showed a major change in the sea level as shown by the oxygen isotope record (Woodruff, 2003) that indicates that sea levels were 100 m above the present sea level during the early Pliocene (5.5–4.5 mya). Such a big decline in the sea level ending at the time we see genetic divergence at 4.59 mya could be a result of an environmental change and indicative of a long dry period (Bohlen et al., 2020; Woodruff, 2003). This subject should still be studied more extensively, but one or more of these possible events were dramatic enough to leave its evolutionary stamp on jellyfish DNA. These changes occurred in the early Pliocene, which was a crucial time for Holarctic carnivoran faunal assemblages (Werdelin & Lewis, 2020). In Africa, one of the first human ancestors, the hominin *Ardipithecus,* appeared—the last common ancestor of humans and living chimpanzees and bonobos (White et al., 2009).

By that estimated time of divergence, 4.59 mya (Figure 6), it seems the eastern and western part of the Mediterranean established their unique marine ecosystems and developed different ecological habitats such as we know today. We suggest that the uniqueness and harsh aquatic ecosystems of the eastern MS developed oligotrophic conditions (Turley et al., 2000) leading to stronger local ecological forces that promoted speciation, genetic pressures and induced higher evolutionary selection than in the other Mediterranean areas. *R*. *pulmo* from the central and western MS (Figures 5 and 6) also exhibited some DNA changes. This may also show that oceanographic processes occur in these areas, which requires further research.

### 4.4 Is the eastern MS *Aurelia* sp. an isolated local population?

The results from the mtDNA COI marker of the *Aurelia* sp. introduced in Figure 7, present, for the first time in the eastern MS, the *Aurelia* sp. (marked with a black rhombus) as an exclusive branch within the evolutionary tree. These results (Figure 7) resemble the data from *R*. *pulmo* (Figures 5 and 6) and emphasize the uniqueness of the local eastern MS species and support the hypothesis that *Aurelia* sp. exhibits concordant phylogeographic patterns (Ramšak et al., 2012) as we see in the *Aurelia* sp. from the East MS as an isolated local jellyfish population. The comparison of the Red Sea, Eilat *Aurelia* sp. (marked with a full circle, Figure 7) data with the eastern MS *Aurelia* sp. from Haifa Bay (marked with a black rhombus, Figure 7), shows clearly that they are distanced from each other. The Eilat *Aurelia* sp. seems to be closely related to the *Aurelia* sp. species from the Pacific Ocean, Papua New Guinea, and the eastern MS *Aurelia* sp. from Haifa Bay is located closer to the Mediterranean species, from Croatia and Slovenia (Figure 7). This picture eliminates the idea that our examined *Aurelia* sp. arrived via Lessepsian migration or that there was any crossbreeding between populations. The *Aurelia* sp. is very common and blooms heavily in the Red Sea of Eilat.

### 4.5 *Phyllorhiza punctata* vector of migration

*Phyllorhiza punctata* sheds light on contemporary processes in the MS through human activity. The evolutionary tree was constructed by combining two rDNA markers, 18S and 28S. Having made this connection of the two genes, the result proved itself and allowed us to have long sequences as a total of 2,844 bp (nucleotide pairing) that enabled good observation and a clear result.

The results suggest that the eastern MS *P*. *punctata* (marked with a black rhombus, Figure 8) from the Israeli shoreline is genetically closer to the Australian *P*. *punctata* than to those from Mexico (Figure 8). We consider the *Phyllorhiza* *sp*. to be indigenous to the Southwestern Pacific (Graham et al., 2003). Based on the results, *P*. *punctata* was recently introduced to the MS as a result of anthropogenic impact on the environment, including introduction via ballast water (Ivanov et al., 2000; Richardson et al., 2009).

## 5 CONCLUSION

Our results inferred that each of the chosen jellyfish has its own characteristic dispersal strategy, although they are quite similar to each other in size and shape. It is common to find them together in swarms, but they exhibit different survival strategies (Dańko et al., 2020, Patrick et al., 2021) that enable them to experience ecological changes differently. The benefit of investigating these similar jellyfish species is that they are each telling a different part of the story of the sea. They shed light on different parts of the geologic history, as we presented in the timetree of the *Rhizostoma* population by marking the divergence time at 4.59 mya. The contemporary processes and anthropogenic impact are presented mostly by *Aurelia* sp. and *P*. *punctata*.

This work demonstrates that the DNA of today's living creatures is of great value to environmental history. We discovered that the characteristics and traits of each species, such as the unique ability and flexibility to fit in and survive in changing environments, specialization in migration, reproduction and feeding strategies, and where the journey of their species' ancestors took them, affect its DNA and provides us with information on the environment in the past and the present. Various genes and different species show us a different resolution for the time period we are investigating. Here, we used the most common DNA markers because they are widely researched, and we were able to acquire extensive worldwide data that helped us accomplish our task. Our findings demonstrated that jellyfish genomes can be used as a phylogeographic molecular tool to trace past events across large temporal scales and revealed that the introduction of invasive species was a result of human activity.

## CONFLICT OF INTEREST

None declared.

## AUTHOR CONTRIBUTION

**Gur A. Mizrahi:** Conceptualization (equal); Data curation (lead); Formal analysis (lead); Funding acquisition (equal); Investigation (lead); Methodology (lead); Project administration (lead); Resources (lead); Software (lead); Supervision (lead); Validation (lead); Visualization (lead); Writing‐original draft (lead); Writing‐review & editing (lead). **Eli Shemesh:** Data curation (lead); Formal analysis (equal); Funding acquisition (equal); Investigation (lead); Methodology (lead); Project administration (equal); Resources (equal); Software (equal); Supervision (supporting); Validation (supporting); Visualization (supporting). **Avia Mizrachi:** Formal analysis (supporting); Methodology (equal); Validation (supporting); Writing‐original draft (equal); Writing‐review & editing (equal). **Dan Tchernov:** Conceptualization (equal); Formal analysis (equal); Funding acquisition (lead); Methodology (equal); Project administration (equal); Resources (lead); Supervision (equal); Validation (equal).

## Data Availability

This genetic data analysis is based on our own collection of samples and on supplementary existing data from GenBank (Geer et al., 2009). DNA sequences list: Accession#: HG931669. GenBank: HG931669.1 Description: *Rhizostoma pulmo* mitochondrial partial COI. Accession#: HG964641GenBank: HG964641.1 *Aurelia* sp. GAM‐2014 mitochondrial partial COI gene for cytochrome oxidase subunit 1. Accession#: HG931673. GenBank: HG931673.1 Description: *Phyllorhiza punctata* partial 18S rRNA gene Accession#: HG931674. GenBank: HG931674.1 Description: *Phyllorhiza punctata* partial 28S rRNA gene SUB9192728 R_pulmo_COI_Israel_D MW698936 SUB9192728 R_Pulmo_COI_Israel_C MW698937 SUB9192728 Aurelia_Israel_COI MW698938 SUB9192728 Aurelia_COI_Eilat MW698939 SUB9192728 R_Nomadica_Israel MW698940
